# A user-driven consent platform for health data sharing in digital health applications

**DOI:** 10.1038/s41746-025-02147-3

**Published:** 2025-11-26

**Authors:** Stefanie Brückner, Akrem Dridi, Aniruddha Deshmukh, Toralf Kirsten, Anne Lauber-Rönsberg, Ronja Riedel, Sven Hetmank, Cindy Welzel, Stephen Gilbert

**Affiliations:** 1 Else Kröner Fresenius Center for Digital Health, TUD Dresden University of Technology, Dresden, Germany; 2 Institute for Medical Informatics, Statistics and Epidemiology, Leipzig University, Leipzig, Germany; 3 Institute of International Law, Intellectual Property and Technology Law, TUD Dresden University of Technology, Dresden, Germany

**Keywords:** Health care, Health policy

## Abstract

Wearable and health app data hold significant potential for healthcare and research, yet fragmented consent mechanisms create challenges for ethical, transparent, and user-controlled data sharing. We describe a centralised consent management system with a structured approach to standardising health data sharing, ensuring regulatory compliance and improving user autonomy. We describe implementation requirements of the system, emphasising regulatory alignment, involvement of app developers, user empowerment, and governance by neutral public bodies.

## Health data as a commodity

We live in an era in which access to data drives innovation and economic growth. The health tech industry has become a multi-billion-dollar economy, producing consumer-facing health apps and wearable devices. Individuals using these technologies generate vast amounts of person-generated health data (PGHD), which holds promise for both personal care (primary use) and secondary use, such as medical research, public health monitoring, and AI-driven healthcare innovations^1^. Yet, PGDH remains underutilised in clinical settings^2^. Real-world health data collected outside traditional care environments could support personalised treatments, early risk detection, and remote monitoring. For example, individuals with diabetes can use self-management tools and continuous glucose monitoring to identify patterns and triggers^3^. This data is more granular and frequent than traditional lab tests conducted during clinical visits, allowing tailored interventions and lifestyle changes. Mental health is another key area. Physiological data from wearables, such as heart rate, sleep and activity information, can predict mood episodes in mood disorder management^4^, support stress management in workplace^5^ and guide treatment plans for people with post-traumatic stress disorders^6^. Beyond personal care, PGHD can complement research databases and support secondary use cases. For example, at-home sensor-collected PGHD offer new insights into symptoms and disease progression^2^. During the COVID-19 pandemic, wearable data donation projects in Germany and the UK contributed to a better understanding of infection dynamics and symptom development^7^.

However, integration into primary care is hampered by fragmented data sources, limited interoperability with electronic patient or electronic health records (EPRs and EHRs), and legal uncertainties around access and consent. Meanwhile, PGHD has become a valuable commodity for tech companies, shaping data-driven business models across both free and paid services^8^. Many users remain unaware of how their data is shared, as disclosures are often buried in lengthy privacy policies, raising ethical concerns about data privacy, autonomy, and digital fairness^9^.

Europe’s regulatory landscape is evolving in response. The European Health Data Space (EHDS) regulation in force since 26 March 2025 and applicable from 26 March 2027, introduces a harmonised framework for health data sharing across EU Member States^10^. While consent according to Art. 9(2) lit. a General Data Protection Regulation (GDPR) remains the central legal basis for primary use of health data, the EHDS regulation, alongside GDPR, will establish mechanisms for secondary use without individual consent^10,11^. The framework introduces national governance structure, health data access, secure processing environments and Member States-specific opt-out mechanisms. Germany, for instance, has introduced the Gesundheitsdatennutzungsgesetz (GDNG, *Health Data Use Act*) and the Digitalgesetz (DigiG, *Digital Act*), mandating electronic patient records for over 70 million public health-insured individuals with the right to opt out^12,13^.

However, it remains unclear whether wellness app providers will be legally required to transfer health data for secondary use under the EHDS, and if so, how user control can be ensured. In a 2022 Joint Opinion, the European Data Protection Board (EDPB) urged that any secondary use of app or wearable data, if included in the EHDS, must rely on explicit, informed consent^14^.

## Is opt-out the level of engagement we want?

For the regulatory framework governing secondary use, the legislation could consider three different options: (1) permitting certain uses by law (e.g., medical research) without individual (i.e., data subject) consent or control, (2) permitting use by law with a data subject opt-out mechanism, or (3) requiring data controllers to obtain consent from data-subjects (opt-in).

The first model, in which secondary use is permitted by law without individual control is often grounded in Article 6(1)(c)(e) or (f) GDPR together with Article 9(2)(i) or (j) GDPR and a specific provision in Union Law or Member State law (see Article 6(3), 9(2)(i) GDPR. The German Cancer Registry, for example, legally requires physicians to enter and report diagnosed cancer cases, thereby creating a fundamental and valuable large-scale population data set for secondary use (Article 7, Sächsisches Krebsregistergesetz, *Saxony Cancer Registry Act*)^15^. Patients may formally object, but this objection only restricts processing of directly identifiable data rather than preventing collection (Article 9, Sächsisches Krebsregistergesetz). Pseudonymised data is then stored and analysed for purposes considered of high public interest, such as public health surveillance, identification of environmental or occupational risk factors, and outcome research. Its legitimacy rests on the presence of strong safeguards, including data minimisation and restricted access, as required by GDPR Article 89. The advantage of this first option is that the legislator can define the purposes of public interest and tasks and ensure representative data. The drawback is the loss of individual informational self-determination.

The second model, legal permission with opt-out mechanisms, provides individuals with more control. Opt-out models can facilitate more data access than opt-in^16^; however, the system depends on users’ inertia to take action or the assumption that the majority of people are willing to share their data. If poorly implemented, this approach can undermine individuals’ autonomy and risks trust erosions. A recent example is the NHS General Practice Data For Planning and Research (GPDPR) initiative in England, which aimed at creating a centralised database of pseudonymised primary care records for research and planning. It faced considerable public backlash and the opt-out of two million patients in 2021 due to lack of transparency, insufficient patient awareness and concerns about commercialisation of data^17^. Using public interest as justification for secondary use often meets resistance when commercial or unknown third parties might be involved.

Additionally, opt-out systems can lack granularity if offering users only a binary choice, which fails to reflect nuanced preferences. For example, a person might agree to share pseudonymised data for public health monitoring but object to its use in commercial AI development. These shortcomings could be partly addressed through improvements such as regular user notifications or tiered opt-outs that allow users to exclude specific data uses. Germany’s new GDNG law includes the requirement for the implementation of such a tiered opt-out model via the electronic patient record. While tiered opt-out models offer more control, there is a risk of reducing user comprehension, specifically if poorly designed. This can lead to errors, unintended participation or users skipping the process all together.

However, even with refinements, opt-out mechanisms assume participation by default and contrast sharply with the third group: consent-based models in participatory medicine, where patients actively engage in their care. In this collaborative model, mHealth technologies support shared decision-making but require a certain level of digital literacy and awareness. Healthcare professionals must be equipped to engage meaningfully with PGHD for successful clinical integration^2^. While technical challenges like missing standards and interoperability persist, an equally important need is a clear legal framework for consent, one that ensures seamless, standardised, and legally sound data access without adding administrative burden.

Although alternative non-consent approaches for clinical data sharing have been proposed, consent will remain central for primary use and many secondary uses of health data, particularly relating to the use of PGHD from apps or wearables. This data is different from clinical data as it is often generated by individuals usually on self-purchased devices, through their personal motivation and with limited clinical involvement. Patients would benefit from a platform that reshapes the control of data sharing by placing user-centricity and transparency at the core. Healthcare providers and secondary data users would benefit from clear and standardised practices to obtain consent. The Standard Health Consent (SHC) platform is one such solution^18^. Unlike opt-out models, SHC empowers individuals to actively manage data sharing from health apps and wearables, enabling informed, granular decisions for both primary and secondary uses through a centralised consent management system. An alternative approach would be to adopt decentralised infrastructures, for examples, blockchain-based architectures, such as the Global Patient Co-Owned Cloud (GPOC) or MedChain^19,20^. These concepts could offer benefits such as enhanced data security, interoperability, and equitable access to healthcare information globally, establishing ownership of data as a human right. Applied to consent, decentralised systems could enhance transparency, traceability, and tamper-resistance while reducing reliance on any single authority. Consent updates, revocations, and data access rights can be encoded into smart contracts, and interactions recorded on permissioned blockchains. However, we focus on a centralised architecture because it aligns with existing structures, such as governance bodies and technical infrastructures, in our target healthcare systems in Germany and comparable EU countries.

## Rethinking digital consent management

Starting from the high-level principle that users of health apps and wearables should have granular control over the data that they choose to share for primary and secondary purposes, we applied speculative design approaches to conceptualise the SHC platform and translate concept into functional prototypes. The digital consent management platform consists of three main components: (1) the SHC Connect integration, which embeds into health apps via iFrame and API, (2) the SHC Management App (SHC App), which can function as a standalone application or as an integration within existing platform ecosystems, such as patient portals or personal health records, and (3) SHC Service for storage and processing (Table 1). All components developed will be released as Open-Source modules at the end of the research project.

**Table 1:** Overview of the key components of the Standard Health Consent Management System: (1) the SHC health app integration, which integrates directly via iFrame and API into health apps, (2) the SHC Consent Management App, which can function as a standalone application or as an integration within existing platform ecosystems, such as patient portals or personal health records, and (3) SHC server for storage and processing

Module	SHC connect	SHC app	SHC service
Function	Enables users to provide and manage consent for health data sharing directly within the embedded health app.	Central access point for users to view, manage, and update consents across all connected health apps under one profile.	Manages user identities, stores consent records, and ensures secure processing and enforcement of consent decisions.
Technical features	Embedded into health apps via iFrame or API to display the SHC interface. Ensures frontend is controlled by SHC to protect user privacy and ensure consistent design. Bi-directional data exchange with SHC Server via API using standardised consent formats.	Angular web application designed for mobile-first use. Initially deployed as a progressive web app, with the option to be packaged as a native app for cross-platform compatibility.	Uses Keycloak for identity and access management. Integrates backend microservices for consent handling, pseudonymisation and secure, scalable data storage using relational databases. Supports interoperability standards such as HL7 FHIR.

**Fig. 1:**
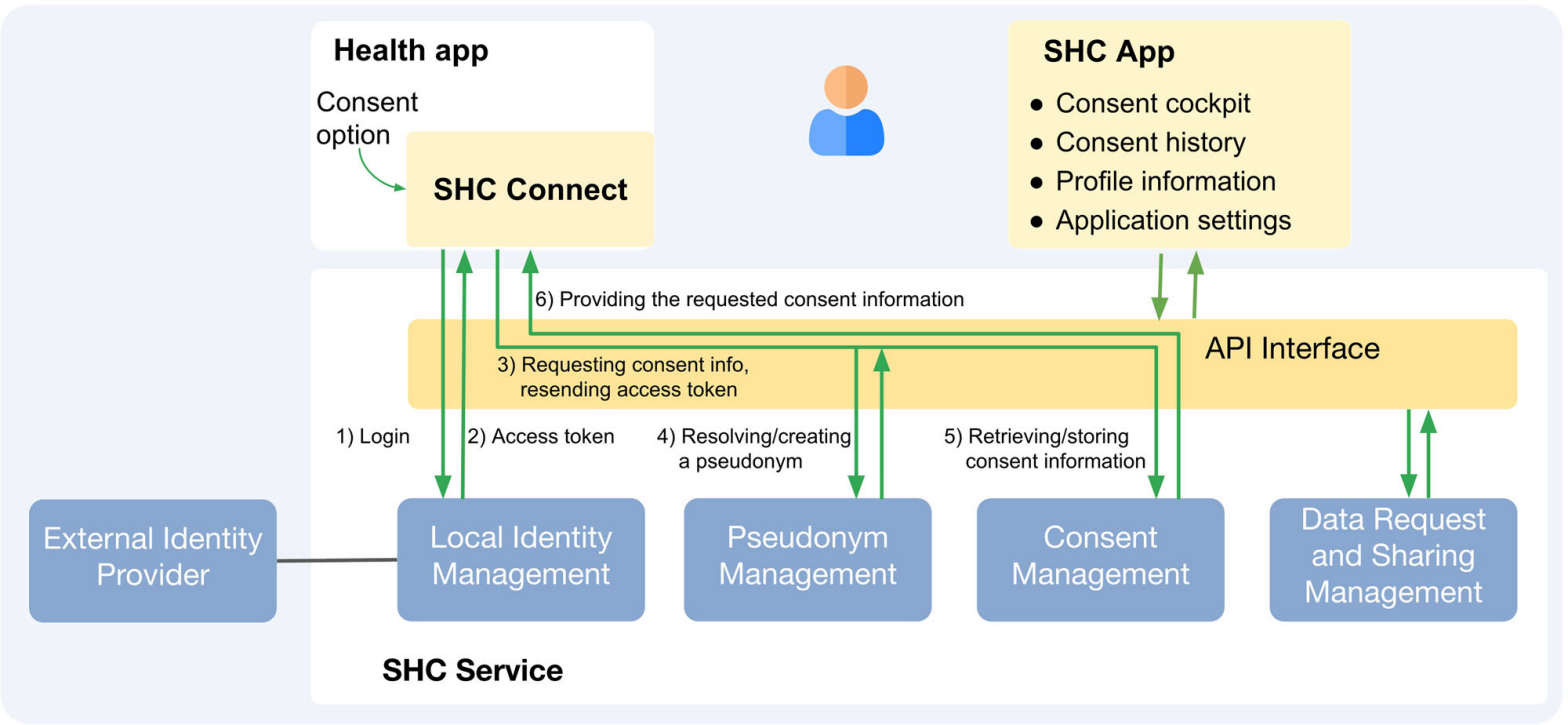
Architecture diagram of the Standard Health Consent System showing the interaction between the user-facing frontend interface and backend services (APIs and management systems). This figure was created with the use of Google Slides.

The high-level system architecture of the SHC components is shown in Fig. 1. At its core is the SHC service, which manages all consent and sharing-related metadata (excluding the actual health data). User authentication and authorisation are managed by the local, open-source identity management system Keycloak, which registers each user and links identification data to a unique user pseudonym^21^. External identity providers, such as health insurers or Germany’s national Health-ID operated by gematik, can be connected to Keycloak and used to authenticate without storing personal identifiers at all.

In practice, the user interacts with the SHC platform for the first time through the health-app embedded SHC Connect module. The interface is optimised for clarity, accessibility and user engagement with adjustments to reading level, text structure, spacing, and the inclusion of visual elements to support comprehension compared to standard legal text. Additionally, accessibility features like screen reading and magnifying text are included to improve usability for people with visual impairments. After being redirect from the health app to the SHC Connect module, users complete registration and authentication. In the next step, the identity management system creates a time-restricted access token and shares it with the SHC Connect module. The SHC Connect module sends this access token to the central SHC Service each time it transfers captured consent data from the SHC Connect module to the SHC service to for each incoming request. Both the SHC Connect module and the SHC App communicate with the SHC service through an API enabled bi-directional exchange of information. Exchanged data formats follow international interoperability standards, particularly HL7 FHIR (Fast Healthcare Interoperability Resources created by Health Level 7) and the FHIR Consent Resource, to ensure compatibility with electronic health/patient records and other digital health services across the EU^22^.

Importantly, the system is designed to protect users’ (i.e., the data subject’s) autonomy and privacy. Each given consent can be changed and withdrawn at any time, and all consent information is managed under a pseudonym. This clear separation between user identity and consent data on the other hand, supports users’ privacy and complies with GDPR.

## Consent flow in an SHC-enabled health app

**Fig. 2:**
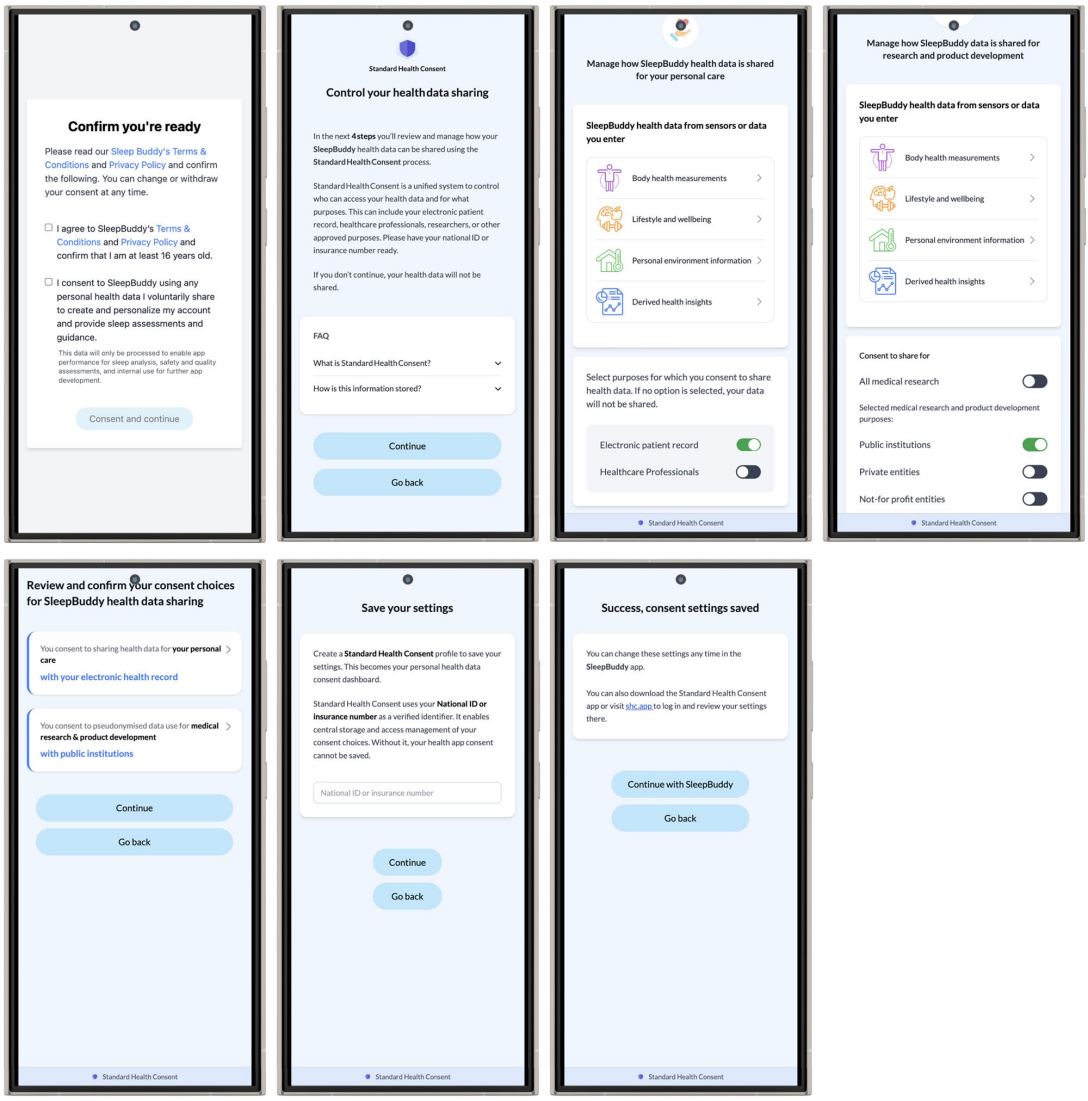
Interfaces of the standard health consent connect. The module integrates via iFrame in health apps and facilitates the consent process.

A full demo of the SHC Connect integration is available under https://shc-demo.vercel.app/connect with key interface elements shown in Fig. 2^23^. When users download an SHC-supported app, they are guided through a structured onboarding and consent process. First, they accept the app’s terms and conditions for its intended use, then set preferences for sharing health data for personal care and secondary use. Users who opt out of SHC will have their data shared only as required for app functionality, or as stated in the SHC contractual framework (Table 2). This prevents apps from misusing the SHC label while engaging in non-consent-based data sharing.

**Table 2:** Overview of contractual terms that govern the SHC platform integration into health applications, covering scope, legal basis, responsibilities, user rights, data retention, liability, termination, and SLAs

Item	Description
Scope of agreement	Defines SHC’s role as the designated consent manager on behalf of the health app. The health app retains full control and responsibility for executing data transfers based on the consent obtained through SHC. Specifies SHC’s scope of services, including supported data types, use cases, and integration requirements. Outlines the health app’s obligations in using SHC Connect for consent-related processes.
Responsibilities of SHC	SHC provides legally compliant tools for consent capture, modification, and revocation. It maintains secure logs of consent actions for audit and regulatory purposes and ensures real-time notification of any updates. SHC is responsible for maintaining service availability as per agreed SLAs.
Responsibilities of the health app	The health app is responsible for ensuring that data is only shared based on valid consent recorded by SHC, and complying with applicable legal obligations. It must ensure that third-party recipients also comply with user consent conditions. The app must act immediately upon withdrawal notifications received from SHC.
User rights and access controls	Users must be able to view, modify, and revoke consent at any time via SHC. SHC provides transparent logs of consent events. The health app cannot override or restrict user choices made through SHC.
Data retention and consent expiry	SHC retains consent records for the legally required duration in compliance with GDPR. Upon withdrawal, SHC notifies the health app immediately to stop any further data use. The health app must implement mechanisms to ensure prompt honouring of revoked consents.
Liability	SHC is liable for any failures in consent management (e.g., incorrect logging, missing consent records). The health app is liable for any unauthorized data sharing that occurs outside of recorded consent or without another legal basis. Each party indemnifies the other for breaches within their respective responsibilities.
Termination and exit strategy	Either party may terminate the agreement in case of non-compliance with consent management obligations. Upon termination, SHC ensures that all consent records are transferred securely to the health app or deleted according to legal requirements.
Commercial terms and SLAs	The SHC service operates under a subscription model (or alternative agreed pricing). SHC guarantees defined uptime (e.g. 99.9%) for consent services and commits to response times for support queries. Support for consent-related issues is included with documented escalation procedures.

It defines the obligations of SHC and health apps in consent management, ensuring transparency, compliance with GDPR, and secure data handling.

To enhance clarity, SHC organises data into four categories: body health measures, lifestyle and well-being information, personal environment data, and health insights derived from these inputs. Additionally, users can access a detailed list of data types collected by the app. For instance, a sleep-tracking app may use all four categories. Tapping a category, such as body health measurements, reveals specifics like sleep time, respiratory rate, age, or weight. This information is provided and updated by the health app.

For personal care, users decide whether to share data with their electronic patient record or specific user-authorised healthcare providers. For secondary use, users can allow or restrict pseudonymised data sharing for research and product development. After reviewing their choices, users confirm their decisions and complete an external identity verification process before their consent is securely stored on the SHC server. Those who skip this step are treated as non-consenting, and no data is shared. In future iterations of the prototype, each screen will be accompanied by context-specific FAQ sections to support users’ understanding. These will explain the concept, governance and intended use of the SHC system and outline the benefits and risks associated with sharing data for personal care or pseudonymised data for secondary use. Educational content will also cover key processes involved in data handling, including pseudonymisation and secure data processing pathways.

## Continuous interaction with consent choices via the SHC app

**Fig. 3:**
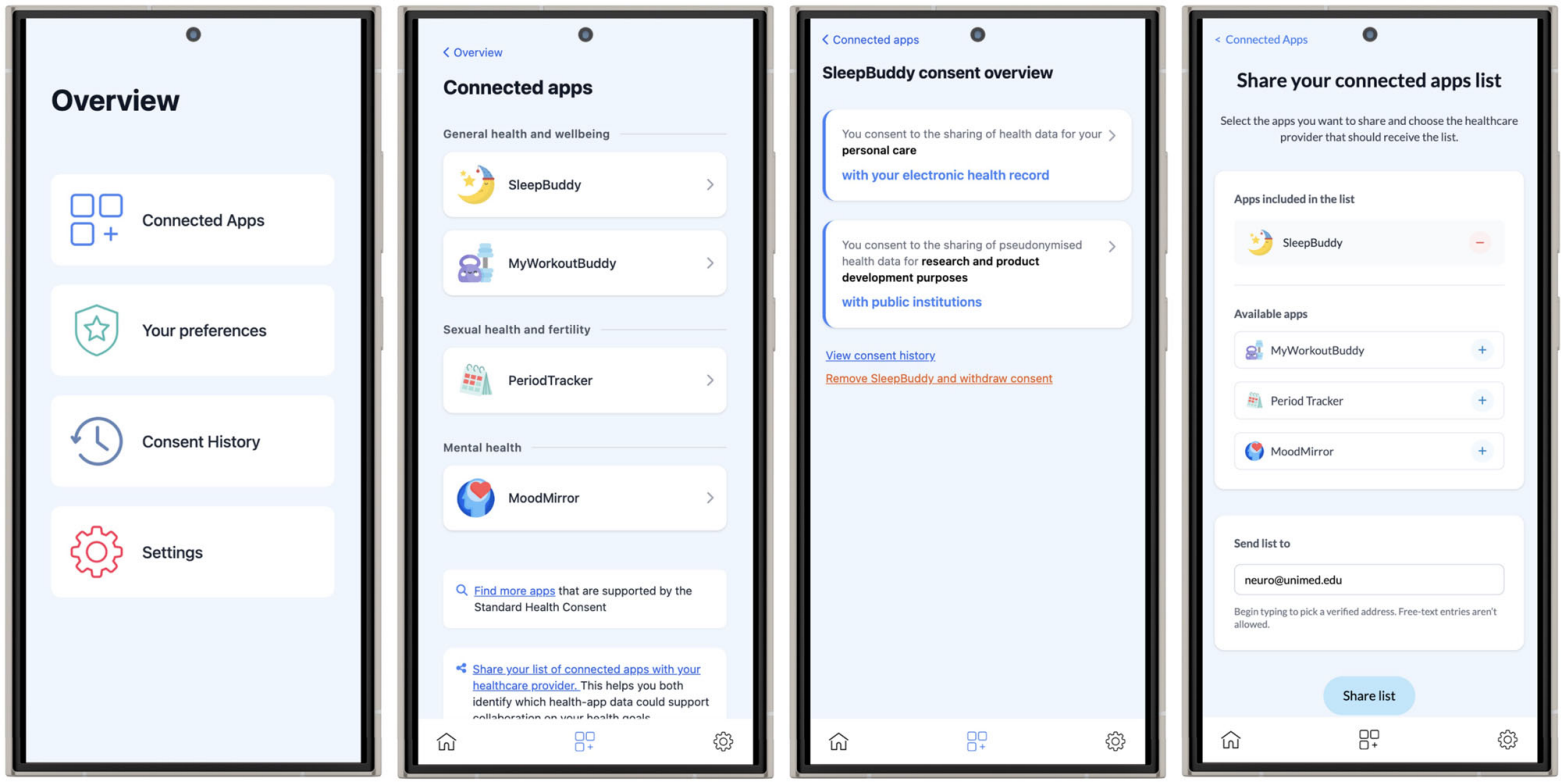
Interfaces of the standard health consent app. Users can view health apps linked to their profile, review consent settings, and modify their data-sharing preferences as needed.

The SHC app provides users continuous access to and dynamic control over their consent choices. The prototype demo is available under https://shc-demo.vercel.app/, with key interface elements shown in Fig. 3^24^. Users can view a central overview of all connected health apps and their current consent settings, with the option to update consents individually or across apps. Each change requires app-specific confirmation to prevent inadvertent or unintended data sharing. To simplify onboarding for new health apps, users can import general preferences, with safeguards in place. A consent history log allows users to track past actions and investigate potential misuse.

The app also includes a registry of SHC-supported apps to help users discover compatible apps, thereby encouraging developers to integrate SHC and increasing adoption.

While the initial onboarding process may seem time-intensive in an era of instant digital services, the added transparency and control justify this deliberate user interface friction. In a digital landscape where speed often outweighs security, SHC prioritises user agency, fostering trust in digital health ecosystems.

## The legal framework under which the SHC could operate

The SHC system acts as an intermediary for managing user consent between app users, health app providers and data requesters. This role is defined by distinct legal relationships, typically governed by contracts. First, the SHC provider will contractually commit to the app users to manage their consents in accordance with their specifications. To fulfil this obligation, the SHC system will require any health app provider that wishes to integrate SHC and outsource consent services to the central SHC system to comply with the SHC contract terms (Table 2), which require that all health-related data sharing is based on explicit user consent obtained through the SHC platform. Finally, the SHC system must ensure, through contractual agreements, that any third parties relying on SHC-based consent for data processing fully comply with the conditions specified by the app users.

Under European law, such relationships must comply with the Data Governance Act (DGA) if SHC facilitates commercial relationships between data subjects and data users^25^. In that case, SHC would qualify as a data intermediation service under Art. 2(11) of the DGA and must adhere to its obligations, including providing fair, transparent, and non-discriminatory access to its services (Art. 12(f) DGA).

The more significant legal challenge lies in compliance with the General Data Protection Regulation (GDPR). Under the GDPR, consent must be informed, specific, and purpose-bound (see Art. 4 (11), Art. 6 (1)(a) and Art. 9 (2)(a) GDPR). This requires that a new consent be obtained for each new processing purpose or recipient. Additionally, users must be informed sufficiently to evaluate benefits and risks, including details on the data controller, processing purposes, and types of data involved. This creates tension with the concept of a standardised consent process. Often, secondary data uses for research are not fully defined at the time consent is gathered. Therefore, data subjects cannot be fully informed in advance, potentially undermining the legal validity of their consent.

There is legal debate over whether the GDPR permits broad consent for scientific research, where exact purposes or recipients may not yet be known. Some interpretations argue that such consent for secondary use may be valid when it refers to general areas of research or categories of recipients at the time point of collection^26^. However, this interpretation has been widely criticised^27^ and the European Data Protection Board (EDPB) emphasises a strict interpretation when sensitive data such as health data is involved^28^. To mitigate this, systems relying on broad consent must implement technical and organisational measures (TOM). One key measure are access boards that review data access requests and allow processing only after a verified application for this approved purpose. However, access boards cannot fully resolve the challenge that, at the time preferences are submitted, not all details about future data processing are known. As a result, the legal admissibility of an SHC platform under EU data protection law may depend on the inclusion of such access boards, similar to those already in place in Germany’s Research Data Centres (Forschungsdatenzentren) (§ 303d SGB V), which are also set to become part of the European Health Data Space (EHDS).

An alternative is Dynamic Consent, where users initially express general preferences without them constituting formal consent. When specific processing purposes arise, users are re-contacted with full information and asked for explicit, use-case-specific consent. While more granular and flexible, this model can be burdensome for users, particularly when their decisions would remain consistent across research contexts with similar benefit and risk factors for the user. In such cases, broad consent may offer better usability without compromising user autonomy, provided safeguards are in place.

In conclusion, a central standardised consent platform such as SHC offers major potential for user-centred data sharing. However, it must be legally and technically designed to meet the stringent requirements of the GDPR and, where applicable, the DGA, including mechanisms that ensure consent remains specific, informed, and revocable, even in dynamic and evolving data-sharing environments. This is the approach we have adopted in the critical design features of the SHC.

## Discussion and conclusion

We argue that a wide-scale adoption of the SHC platform would represent a major step toward a more transparent, user-centric approach to health data sharing. By replacing fragmented consent experiences with a single, structured interface, SHC empowers individuals to manage health data sharing across apps in a consistent and informed way. While SHC itself does not execute data transfers, it offers a trusted consent infrastructure, ensuring that sharing only occurs under clear user-defined conditions. Legal responsibility for secure and lawful data use remains with the individual health applications. In the future, national Health Data Access Bodies (HDAB), required under the EHDS Regulation will have an important governance and infrastructure role, including the processing data requests and issuing of permits as well as the provision of closed secure environments for data access (see Art. 57 (1) EHDS regulation)^10^. Health apps that collect and process electronic health data must implement robust technical and organizational safeguards to ensure compliance with the granted consent and regulatory requirements. HDAB will be responsible for verifying that data holders meet trustworthiness criteria, maintaining compliance with regulatory obligations, and continuously auditing access mechanisms. In addition, it will authorise data access only after a reviewed request has been found to match a verified and approved purpose. By establishing these multilayered safeguards, the SHC platform, alongside EHDS structures, promotes a secure, transparent, and user-centric model of health data sharing, ensuring that individuals retain control over their information while enabling valuable secondary use for research and public health advancements.

In a digitally connected healthcare system, where data is expected to flow seamlessly between patients, providers, and researchers, a robust consent infrastructure is essential. SHC not only supports personalisation and control, but also opens the door for integrating feedback mechanisms that foster user trust and engagement. Real-time audit trails and usage notifications can help inform users not just of intended, but of actual data use. Future integrations with technologies such as blockchain-based smart contracts or de-identified tokens could further enhance transparency^29^.

The design of SHC was informed by stakeholder and user feedback, and shaped by key implementation decisions across system architecture, governance, technical integration, and digital literacy. Table 3 summarises these considerations, which will guide future iterations. In a deliberate choice, we developed the SHC system as a centralised architecture. Centralisation enables direct assignment of responsibility for consent logic, secure identity management, and consistency across health apps. These properties are priorities for accountability, safety, timely deployment in app and consistent user experience. However, this model also introduces limitations, including the reliance on a single infrastructure and reduction in scalability. Ultimately, the SHC and decentralised models share the core principles of user control and empowerment and future work could explore decentralised or hybrid approaches for SHC.

**Table 3:** Key questions during SHC platform development and implemented solutions

Design domain	Summary of trade-offs
System structure	Centralisation ensures improved control over identity and consent logic, easier enforcement of policies and consistency across apps. However, it reduces scalability and increases dependency on a single infrastructure.
Hosting and oversight	Public or mandated hosting by non-profit, governmental or institutional body ensures business neutrality and user trust, prioritising user empowerment over data monetisation. It may involve slower governance and funding complexities compared to private operators.
Health app integration	Initial integration via iFrame enables fast setup without requiring app redesign and ensures consistent user experience across platforms. However, it comes with performance and security limitations, making a future transition to API and Webhook integration necessary to support scalability, real-time updates, and improved system resilience.
Digital literacy level	Focusing on users with basic literacy improves usability insights and real-world relevance, but limits early feedback from edge user groups with higher or lower literacy.

A key challenge is maintaining user engagement without creating cognitive or administrative burden. Consent processes must be intuitive, yet meaningful, enabling users to exercise real control without being overwhelmed. Future research should explore how users interpret consent granularity, what levels of complexity they find acceptable, and how preferences evolve over time.

Legitimate concerns are raised about the low participation rates in consent-based systems and risk of mass withdrawal in opt-out. Both situations may reduce sample size and introduce bias, as individuals who decline consent or opt out might differ systematically from those who remain in the dataset. Compared to non-consent/no opt-out systems, lower sample sizes are to be expected leading to lower statistical power of analysis^30^. However, these risks can be mitigated through effective public engagement strategies, including broad awareness campaigns as well as tailored outreach to specific groups, such as minority populations who may have lower baseline trust or digital access. Information must be provided in accessible, relevant and trustworthy formats. A successful example is the “All of Us” Research Program by the National Institute of Health (NIH, USA), which enrolled over 650,000 participants, many from historically underrepresented groups, by partnering with community organizations, offering multilingual materials, and providing a transparent dashboard that shows how participant data is used. Additionally, statistical methods like raked weights can be applied to iteratively reweight the sample for specific variables (e.g., age, gender, ethnicity and others) to match the variables distribution in the reference population^31^. This enables statistically significant and relevant analyses without the need to include the entire population, as working with representative samples is a well-established and accepted practice in scientific research. Crucially, such engagement must be accompanied by a clear and enforceable data governance framework for secondary use, including transparency around data access by commercial entities to prevent public outcries as experienced in the UK^17^. Together, these measures can help establish a renewed social contract grounded in genuine public trust, informed decision-making, and voluntary participation.

The EU AI Act introduces transparency obligations for data used in training high-risk AI systems (Article 10), including requirements to document data sources, processing methods, and representativeness^32^. The SHC platform can support these obligations by offering user-specific consent records and auditable provenance of data shared from health apps. This strengthens legal and ethical accountability for AI development, reinforcing user trust and lawful data governance. This could underscore EU’s strong stance as a global leader in AI regulation, standing in harsh contrast to large-scale non-consent and non-transparent data scraping for Large Language Model developments we have seen so far^33^.

Based on the analysis of relevant regulatory frameworks and the development of the SHC platform, we propose a set of key recommendations as outlined in Table 4 to support the implementation of a standardised digital consent infrastructure. The recommendations summarise the importance of regulatory alignment, neutral governance, and technical interoperability to support a trusted consent infrastructure. User empowerment through granular, revocable consent and transparency mechanisms is essential for building public trust. Addressing consent bias and ensuring inclusive participation are critical for equitable and representative health data use.

**Table 4:** Recommendations for the implementation of standardised consent management

Recommendation	Stakeholder	Rationale
Mandate standardised consent for apps participating in EHDS data sharing	EU and national legislators	Promotes uniform, privacy-preserving consent aligned with EHDS goals
Governance and oversight by public or mandated bodies	SHC provider, Ministries of Health, HDABs, national digital health agencies	Ensures neutrality, avoids conflicts of interest, builds trust
Tiered consent to express preferences for secondary use	App developers, SHC provider	Supports user autonomy and transparency
Dynamic consent dashboards and continuous communication	SHC provider	Empowers users with continuous control; meets GDPR revocation and auditability requirements
Public registry of SHC compliant apps	SHC platform providers. national digital health agencies	Increases transparency and incentivises responsible app design
EHDS interoperability	EU and national digital health agencies	Ensures EPR/EHR and infrastructure integration; future-proofs compliance
Bias monitoring for consent decisions; outreach to non-consenting groups	Ministries of health, patient advocacy groups, local communities	Prevents exclusion and supports equity in data use

In conclusion, standardised consent management offers a transformative approach to health data sharing from apps and wearables. Platforms like SHC enable dynamic, transparent, and user-driven consent processes. They create a scalable foundation for ethical, secure, and interoperable data sharing, balancing individual autonomy with the needs of healthcare and research. The future of health data governance will depend not only on legal frameworks, but also on empowering tools that make consent both meaningful and actionable for every user.

## Data Availability

No datasets were generated or analysed during the current study.
